# Intermediate between Idiopathic Hypereosinophilia and Chronic Eosinophilic Leukemia: A Report of Two Hypereosinophilic Cases with Possible Novel Molecular Mutations

**DOI:** 10.1155/2021/1142124

**Published:** 2021-08-31

**Authors:** Jui Choudhuri, Mohammad Eskandari, Yang Shi, Yanhua Wang

**Affiliations:** ^1^Department of Pathology, Montefiore Medical Center/Albert Einstein College of Medicine, 111 East 210^th^ Street, Bronx 10467, NY, USA; ^2^Department of Pathology, Altru Health System, 1200 S. Columbia Rd., Grand Forks 58201, ND, USA

## Abstract

To distinguish a reactive eosinophilia from its malignant counterpart is challenging. Establishing clonality of the eosinophils is crucial and considered the determining factor for establishing a diagnosis. Cases of hypereosinophilia without clear reactive etiologies, no evidence of end-organ damage, normal cytogenetics, and no molecular mutations are termed as “Idiopathic Hypereosinophilia (IHE).” For cases which lie between the spectrum of chronic eosinophilic leukemia (CEL) and IHE, identification of underlying molecular abnormalities might be helpful in better understanding the disease process and prognosis. Here, we report two cases of hypereosinophilia in which five possible novel molecular mutations were identified by targeted next-generation sequencing (NGS) analysis. They were FBXW7, KM2A, TCF3, ERBB4, and MET. With multiple genetic mutations, these cases could be classified as chronic eosinophilic leukemia. Both these young patients responded well to steroid therapy. While targeted NGS is a useful tool in identifying new molecular mutation associated with hypereosinophilia, our cases raise the question of further investigating this entity and if there is a possibility of an intermediate category lying between the spectrum of CEL and IHE. Defining hypereosinophilia with clonal molecular abnormality as a malignant process may need to be revisited. Even though attempts are being made to identify mutations in IHE, it might be more significant clinically to differentiate them based on response to steroid therapy and prognosis.

## 1. Introduction

Idiopathic hypereosinophilic syndrome (HES) is defined as eosinophilia (>1.5 × 10^9^/L) that persists for at least 6 months with no identifiable causes and is associated with end-organ damage and dysfunction in multiple organs [1–3]. Chronic eosinophilic leukemia (CEL) is a myeloproliferative neoplasm with autonomous and clonal proliferation of eosinophil precursors resulting in increased numbers of eosinophils in the peripheral blood (PB), bone marrow (BM), and/or peripheral tissues [4]. Myeloid and lymphoid neoplasms with eosinophilia, in contrast, are associated with gene rearrangements of PDGFRA, PDGFRB, FGFR1, or PCM1-JAK2 [4]. These neoplasms have different clinical and hematological findings, influenced by the partner genes involved [5]. HES and CEL are diagnoses of exclusion and rendered after ruling out reactive processes and hypereosinophilia associated with myeloid and lymphoid neoplasms such as acute myeloid leukemia (AML), myelodysplastic syndrome (MDS), and systemic mastocytosis (SM) [4, 6].

Distinguishing HES and CEL is sometimes challenging. CEL differs from HES by showing clonality and/or increased blasts by the 2016 World Health Organization (WHO) criteria [4]. The clonality in these cases is mainly evaluated by conventional karyotyping and/or identifying specific mutations for myeloid neoplasms. In cases of hypereosinophilia without clear reactive etiologies, no evidence of end-organ damage, normal cytogenetics, and no molecular mutations, the term “idiopathic hypereosinophilia (IHE)” is warranted [4]. Recent studies on IHE patients have identified mutation in 28–53% of patients in various studies [7, 8]. The range of gene mutation and most frequently mutated genes has varied in studies.

Here, we reported two cases of persistent hypereosinophilia in which possible novel gene mutations were identified and these have not been reported previously in literature; however, the response to treatment made the diagnoses challenging.

## 2. Case Presentation

**Figure 1:**
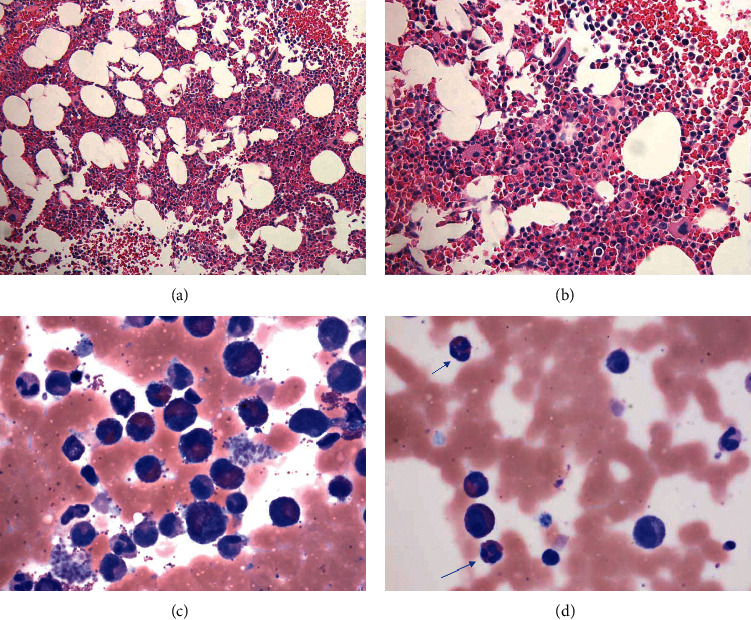
Bone marrow biopsy in a 17-year-old female with no significant past medical history. (a) H&E (400X magnification) section of the bone marrow biopsy, with trilineage hematopoiesis and a significant increase in eosinophils. (b) H&E (400X magnification) section of the bone marrow biopsy. (c), (d) Aspirate at high power (1000X magnification) showing an increase in mature eosinophils with occasional trilobed nuclei (arrow) and no increase in blasts.

The first patient, a 17-year-old female, with no significant past medical history, presented with 1.5 months of facial angioedema, shortness of breath, chest pain, pleural effusion, acne-type rash, and visual loss. There was no history of recent medication prior to onset of symptoms, and a complete blood count was significant for eosinophilia. C1 esterase quantitative was normal. She had received antibiotics and antihistaminic at another facility. Imaging demonstrated diffuse lymphadenopathy and bilateral pleural effusions. Tuberculosis, autoimmune etiology including hereditary angioedema, rheumatologic etiology including sarcoidosis or systemic lupus erythematosus (SLE), and an oncologic process including lymphoma were excluded. 81% eosinophils were identified in the pleural fluid. Bone marrow biopsy was normocellular with trilineage hematopoiesis and approximately 30% eosinophils with some trilobed and multilobated forms. There was no increase in blasts, and some small hypolobated megakaryocytes were present (Figure 1). She was treated subsequently with steroid (prednisone, 60 mg/day and weaned gradually over seven months) alone, and the eosinophil counts dropped rapidly. Her overall eosinophil count remained slightly above the normal range afterwards. She still had some vision and sinus symptoms, but other symptoms resolved. Her absolute eosinophil count declined from 12.9 k/ul (normal 0.1–0.3 k/ul) to 0.4 k/ul over the course of the treatment and continued to remain within the normal range at one-year follow-up.

**Figure 2:**
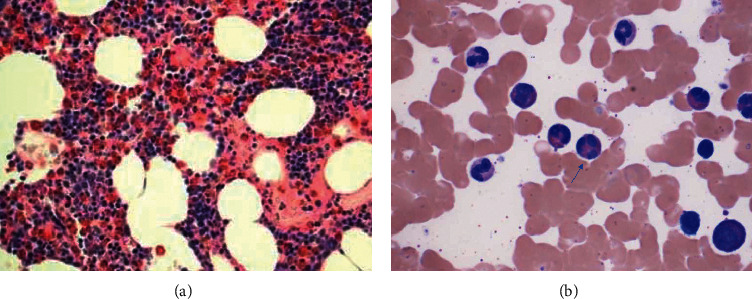
Bone marrow biopsy in a 33-year-old female with a past medical history of multiple sclerosis. (a) H&E (400X magnification) section of the bone marrow biopsy, with trilineage hematopoiesis and a significant interstitial increase in eosinophils. (b) Aspirate at high power (1000X magnification) showing mature eosinophils with rare trilobed nuclei (arrow) and no increase in blasts.

**Figure 3:**
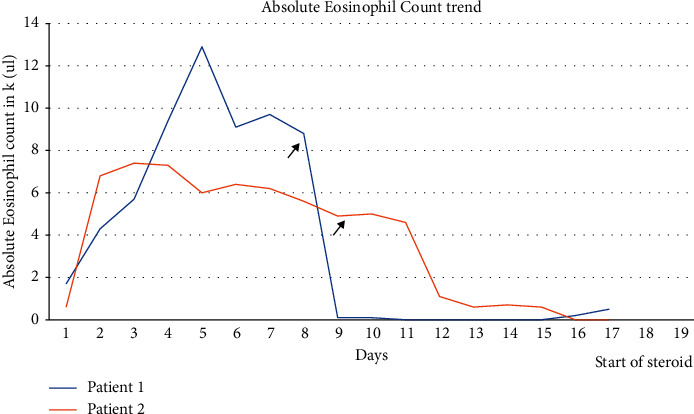
Trend of absolute eosinophil counts (k/ul) of both patients over time following treatment with steroid. Blue line: patient 1; orange line: patient 2. The arrows indicated steroid usage.

The second patient was a 33-year-old female with past medical history of multiple sclerosis, who presented with abdominal pain for three weeks and was diagnosed and treated for colitis. She was noted to have ∼60% eosinophils on complete blood count. Infection and nonhematological malignancy were ruled out. A pleural effusion was noted incidentally on an abdominal X-ray. A thoracentesis was performed, and the pleural fluid analysis was notable for 86% eosinophils. A bone marrow biopsy revealed a relatively normocellular marrow (overall 60% cellularity) with trilineage hematopoiesis and marked increase in eosinophils (25% on aspirate); rare eosinophils showed abnormal lobation (trilobed forms) (Figure 2). The patient was placed on intravenous methylprednisolone 70 mg per day. Her eosinophil count decreased, from 7.4 k/ul to 0.1 k/ul, and she was discharged from the hospital 4 days later (Figure 3). She did have a recurrence two years later with an eosinophil count of 3.3 k/ul but responded promptly to steroid treatment and has been asymptomatic since then with normal eosinophil count.

Both patients showed normal karyotype and were negative for PDGFRA/B or FGFR1 rearrangements by cytogenetics/fluorescence in situ hybridization methods. Next-generation sequencing (NGS) for both showed different mutations (allele burden >5%) with no overlapping genes. The first patient showed four mutations: FBXW7 (c.566_567delAAinsGT; p.K189S), KMT2A (c.3634 + 4G > A), TCF3 (c.1357G > A; p.A453T), and TCF3 (c.635C > T; p.A212V), and the second patient showed three mutations: ASXL1 (c.2866C > T; p.L956F), ERBB4 (c.644A > C; p.E215A), and MET (c.467C > T; p.S156L). While ASXL1 and FBXW7 have been reported previously [7, 8], the other mutations identified, KM2A and TCF3 in the first patient and ERBB4 and MET in the second patient, have not been reported previously in patients with eosinophilia to the best of our knowledge; however, they are categorized as tier 1 according to the Catalogue of Somatic Mutations in Cancer (COSMIC) [9] (Table 1).

## 3. Discussion

Eosinophilia can coincide with different nonhematologic and hematologic disorders and carries potential for end-organ damage. The normal count of eosinophils in the peripheral blood is 3–5% which corresponds to an absolute eosinophil count (AEC) of 350–500/mm^3^ [10]. In the clinical setting, eosinophilia can be divided into mild (500 < AEC < 1500), moderate (1500 < AEC < 5000), and severe (AEC > 5000) [10, 11]. Diagnosis of primary eosinophilia relies on multiple steps, including exclusion of secondary etiologies of eosinophilia, as well as morphologic review of the peripheral blood and bone marrow, cytogenetics, FISH, flow cytometry, and clonal T-cell gene rearrangement studies to detect histopathologic or clonal evidence of an acute or chronic myeloid or lymphoproliferative disorder.

The 2008 World Health Organization defined a semimolecular classification scheme of disease subtypes including myeloid and lymphoid neoplasms with eosinophilia and abnormalities of PDGFRA, PDGFRB, or FGFR1, chronic eosinophilic leukemia-not otherwise specified (CEL-NOS), lymphocyte-variant hypereosinophilia, and idiopathic hypereosinophilic syndrome (HES), which was categorized as a diagnosis of exclusion [12]. The 2016 WHO classification revision added a provisional entity of PCM1-JAK2 rearrangement neoplasms [4, 13]. Mattis et al. in their recent work have described the algorithm for diagnosis of IHE/HES and CEL and have emphasized that IHE and HES are two distinct entities separated by organ damage and the terms should not be used interchangeably [3].

While various studies have tried identifying mutations for IHE, few common mutations have been identified. Wang et al. in their work on HES found 28% of patients carrying somatic mutations and with the most frequent being ASXL1 [7]. Their study showed that mutations frequently affect genes involving DNA methylation and chromatin modification. HES patients with verified mutations showed several clinical, laboratory, and bone marrow histomorphologic features resembling those of CEL-NOS. Their findings suggested that molecular and genetic status was critical for leukemogenesis, most likely to be correlated with morphologic abnormalities; integrating morphological assessment into the interpretation of genetic data might aid to distinguish a true neoplasm from nonneoplastic hypereosinophilia [7, 14]. Another study by Lee et al. identified 53% patients with IHE/HES to have somatic mutations and the most frequent was NOTCH1, having hidden T-cell malignancy potential. They saw more dysplastic eosinophils in patients with mutations and concluded that somatic mutations are likely to be associated with clonal proliferation of eosinophils, possibly including myeloproliferative neoplasm [8] (Table 2).

Andersen et al. performed whole exome sequencing and genome-wide methylation analysis on purified eosinophils from patients with idiopathic HES. They found somatic missense mutations in cancer-related genes in three patients. The genes included spliceosome PUF60 and the cadherin gene CDH17 [15]. In our patient population, we identified two of 16 patients diagnosed with IHE having possible somatic mutations (unpublished data). A germline mutation is considered to have an allele frequency of 50 to 100% [16]. A somatic mutation is usually present with a lower allele frequency because it is not present in all cells [17]. Four mutations were identified in the first patient's sample, and three mutations were identified in the second patient's sample. Most likely, it is because the samples contained a very high percentage of neoplastic eosinophils, and these eosinophils showed heterozygous mutations. Therefore, the VAF is approximately 50%. It is less likely that the patients had three or four germline mutations at the same time. However, germline mutations cannot be completely ruled out due to lack of oral epithelial cell controls. The next-generation sequencing panel with a larger array of mutations revealed novel mutations which have not been identified previously. These genes include KMT2A, TCF3, ERBB4, and MET. Three of these mutations were missense, including ERBB4 (c.644A > C; p.E215A), MET (c.467C > T; p.S156L), and TCF3 (c.1357G > A; p.A453T and c.635C > T; p.A212V). The KMT2A gene has a splice-region alteration (c.3634 + 4G > A).

KMT2A is also known as myeloid/lymphoid or mixed-lineage leukemia (MLL), a gene which encodes a transcriptional coactivator playing an essential role in hematopoiesis. Mutation of this gene has been seen in acute lymphoblastic leukemia and acute myeloid leukemia [18]. A variant of KMT2A (MLL) was also reported in Wiedemann–Steiner Syndrome (WSS), an abnormality in facial and skeletal growth, as well as intellectual development delay [19]. KMT2A can translocate to many different partners; some translocations might be associated with eosinophilia; however, the exact mechanism has not been elucidated [20].

TCF3 encodes a member of helix-loop-helix transcription factors. It is required for B- and T-lymphocyte development. Deletion or mutation of this gene may play an important role in lymphoid malignancies. It has been reported involving in several chromosomal translocations in different leukemias or lymphomas, such as with PBX1 in *t*(1; 19) reported in pre-B acute lymphoblastic leukemia, with TFPT in *t*(19; 19) seen in pediatric leukemia and with ZNF384 in *t*(12; 19) in acute leukemia [21–23]. However, its association with eosinophilia has not been reported yet.

ERBB4 is a member of the tyrosine kinase family and the epidermal growth factor receptor subfamily. It maintains a critical role in regulating cell proliferation and differentiation, as well as cell migration and organogenesis. Mutations in this gene have been associated with different malignancies, including sarcoma, cancer, and lymphoma [24–27]. Different expression patterns of EGF, EGFR, and ERBB4 in the nasal polyp can be associated with an increase of eosinophilic infiltrate, indicating a cytokine role related to ERBB4 [28]. However, its role in pathologic eosinophilia is not fully understood. More cases with this mutation might be needed to characterize its mechanism.

MET is another member of the receptor tyrosine kinase family. It plays a role in cellular survival, embryogenesis, and cellular migration and invasion. Mutations of the gene with amplification and overexpression have been found to be associated with several human malignancies, such as papillary renal cell carcinoma, hepatocellular carcinoma, and various head and neck cancers [29–31]. However, the relationship of MET mutation with eosinophilia has not been reported yet.

According to the 2008 WHO classification, our two cases are classified as IHE. However, the 2016 WHO revision stated clearly that idiopathic hypereosinophilia is defined as eosinophilia with no evidence of eosinophil clonality [4, 32]. If multiple mutations are considered evidence of eosinophilic clonality, then our cases are classified as CEL, not otherwise specified, according to the 2016 WHO revision. Both our patients were young, with bone marrow involvement but responded well to steroids and did not require chemotherapy. They did not have any organ damage. This leads to a dilemma in classifying them as CEL which is a rare, aggressive disease with a high risk of acute transformation [33]. Patients with hypereosinophilia that mostly involves the bone marrow might present with anemia and thrombocytopenia, while patients with hypereosinophilia that mostly involves extramedullary organs might present with symptoms related to cytokine release, such as sinusitis, gastroenteritis, neuritis, pleural effusions, abdominal pain, and shortness of breath. The two scenarios might have different disease mechanisms and presentations. Treatment options include high-dose steroids followed by other agents such as hydroxycarbamide, interferon-alpha, and imatinib, for corticosteroid-resistant or corticosteroid-sparing cases. Patients with bone marrow disease might benefit from chemotherapy and bone marrow transplant.

In the previous literature, idiopathic hypereosinophilia has been reported as a rare chronic condition which can sometimes be fatal in the older age group and prognosis depends on organs involved [1, 34]. Our cases bring up the question if these should be classified based on mutations or good response to steroid, also raising the possibility of an intermediate category with clonality and yet excellent prognosis and responsiveness to steroid. These cases highlight that, for IHE patients, even though attempts are being made to identify mutations, the relevance of mutations is still not well understood, and it might be imperative to investigate them further or classify clinically for patients and clinicians. Further studies will help clarify both the overlapping and unique features of these two entities and to understand if there are entities lying within this spectrum which can be classified separately. This information will be extremely relevant for clinicians, pathologists, and patients.

## 4. Conclusions

In summary, we present two cases of IHE with possible novel mutations identified on NGS and that have not been previously reported. Some of these mutations have undetermined clinical significance at this point. However, the excellent response to steroid for both patients make it important to study this disease entity and investigate it based on both either molecular mutations or clinical profile.

## Figures and Tables

**Table 1:** Demographic and mutation analysis result in two cases of idiopathic hypereosinophilia.

Age (years)/gender	Mutations	Nucleotide change	Allele frequency (%)	Tier	Karyotype/FISH analysis
17/F	FBXW7	c.566_567delAAinsGT; p.K189S	45	1	46, XX
	KMT2A	c.3634 + 4G > A	48	1	No rearrangement of PDGFRA (4q12) and PDGFRB (5q31)
	TCF3	c.1357G > A; p.A453T	51	1	FGRFR1 (8p11)
	TCF3	c.635C > T; p.A212V	51		MDS/MPN FISH panel: negative^*∗*^

33/F	ASXL1	c.2866C > T; p.L956F	47	1	46, XX
	MET	c.467C > T; p.S156L	49	1	FGRFR1 (8p11)
	ERBB4	c.644A > C; p.E215A	53	1	No rearrangement of PDGFRA (4q12) and PDGFRB (5q31)

*∗*FISH panel for MDS/MPN includes the following probes: EGR1, D7Z1, D7S486 (7q31), D8Z2 (8 CEN), KMT2A (MLL), TP53, D17Z1 (17CEN), D20S1157 (20PTEL18), D20S108 (20q12), PDGFRA, PDGFRB, CSF1R, FGFR1, CEP 9, ABL1, BCR, D13S319 (13q14.3), and LAMP1 (13q34) (Vysis FISH Probes, Abbott Molecular).

**Table 2:** Mutations identified in the literature.

Author	Frequency of mutation	Genes involved
Wang et al.	28%	ASXL1, TET2, EZH2, SETBP1, CBL, NOTCH1 DNMT3A, NRAS, JAK2 exon 13, and GATA2
Lee et at.	53%	NOTCH1, TP53, TET2, EZH2, FLT3, IKZF1, ITPKB, SAMHD1, SF3A1, STAG2, ZMYM3, CDKN2A, ATRX, DIS3, BRD4, CARD6, GATA2, NFKBIE, SMC1A, ASXL1, ATM, BIRC3, CBL, CCND1, CEBPA, FAM46 C, FAT4, FBXW7, GATA1, MAPK1, MPL, NF1, PRKD3, PRPF40 B, RUNX1, SCRIB, SF1, SF3B1, SH2B3, SMC3, WT1, PTEN, MED12, BCOR, CSF1R, CSF3R, DNMT3A, EGR2, HIST1H1E, JAK2, LRP1B, POLG, PRKD3, PRPF40 B, RB1, SETBP1, SMARCA2, SMC3, TGM7, U2AF2, and ZRSR2
Choudhuri et al.	—	FBXW7, KMT2A, TCF3, ASXL1, ERBB4, and MET

## Data Availability

The data used to support the findings of this study are restricted in order to protect patient privacy. Data are available for researchers who meet the criteria for access to confidential data.
